# Errata

**Published:** 1887-12

**Authors:** 


Errata.—In our report of the discussion upon Hay Fever, at last meeting of I. H. A., *Colchicum* was given for *Causticum*. See page 322, seventh line from bottom.

In the lecture on Sulphur, in October number, page 375, line sixteen from the top, by reason of error in punctuating the Graphite symptom, "Outer surface of nose becomes filled with black pores" is made to read as if it were a symptom of Euphrasia. A period should *precede* "Outer," which should begin with a capital letter. Page 376, line seventh, a period should be placed after "useful." *When* begins a new sentence.

November number, page 403, line 9 from the bottom, for rugged read *ragged*. Page 405, line 19 from top, for panting read *pouting*.

